# Expression and role of PGP, BCRP, MRP1 and MRP3 in multidrug resistance of canine mammary cancer cells

**DOI:** 10.1186/1746-6148-9-119

**Published:** 2013-06-17

**Authors:** Karol M Pawłowski, Joanna Mucha, Kinga Majchrzak, Tomasz Motyl, Magdalena Król

**Affiliations:** 1 Department of Physiological Sciences, Faculty of Veterinary Medicine, Warsaw University of Life Sciences - WULS, Nowoursynowska 159, 02-776, Warsaw, Poland; 2 Department of Large Animal Diseases with Clinic, Faculty of Veterinary Medicine, Warsaw University of Life Sciences – WULS, Nowoursynowska 100, 02-797, Warsaw, Poland; 3 Department of Animal Environment Biology, Faculty of Animal Sciences, Warsaw University of Life Sciences - WULS, Ciszewskiego 8, 02-786, Warsaw, Poland

**Keywords:** Multidrug resistance, Chemotherapy, Canine mammary cancer, Vinblastine, Cisplatin, Cyclophosphamide, PGP, BCRP, MRP1, MRP3

## Abstract

**Background:**

In both women and female dogs, the most prevalent type of malignant neoplasm is the spontaneous mammary tumor. In dogs, half of these are malignant. The treatment of choice for the canine patients is surgical mastectomy. Unfortunately, it often fails in high-risk, locally invasive mammary tumors as of during the time of the surgery the micro-metastases are present. Moreover, there are neither large studies conducting to prove of the benefit from the chemotherapy in dogs nor established chemotherapy treatment protocols available. Additionally, the effectiveness of each individual chemotherapeutic agent and drug resistance of canine mammary cancer have not yet been characterized. That has become the aim of our study, to assess the expression of PGP, BCRP, MRP1 and MRP3 in canine mammary cancer cell lines and to investigate their role in cancer resistance to vinblastine, cisplatin and cyclophosphamide with using RNAi approach.

**Results:**

The results suggested that in canine mammary cancer, the vinblastine efflux was mediated by PGP and MRP1 proteins, cisplatin efflux was mediated by all four examined efflux pumps (PGP, BCRP, MRP1 and MRP3), whereas cyclophosphamide resistance was related to BCRP activity. RNAi silencing of these efflux pumps significantly decreased IC50 doses of the examined drugs in canine mammary carcinoma cells.

**Conclusions:**

Our results have indicated the treatment of cells involving use of the siRNA targeting efflux pumps could be a beneficial approach in the future.

## Background

In both women and female dogs, the most prevalent type of malignant neoplasm is the spontaneous mammary tumor. In dogs, half of these are malignant. There is a threefold incidence in the canine, with middle aged, non-spayed female dogs being the most affected [1,2]. The early ovariectomy is thought to reduce the risk of mammary cancer development [3], however, the high morbidity and mortality rate due to ineffective treatment strategies makes this problem as being still actual. The treatment of choice for the canine patients is surgical mastectomy. Unfortunately, it is often recognized - unsatisfactory as in case of invasive cancer at the time of surgery the micro-metastases are present. Moreover, clinical studies reveal that after regional mastectomy 77% of dogs developed a new malignancy [4]. There are neither large studies conducting to prove to the benefit of chemotherapy in dogs nor established chemotherapy treatment protocols available. Moreover, the effectiveness of each individual chemotherapeutic agent and drug resistance of canine mammary cancer have not yet been characterized. The available data is conflicting, possibly due to the small number of studies performed, or the insufficient number of dogs used in these studies. Some veterinarians adapt human chemotherapy protocols; however these treatment modalities are often unsatisfactory due to similarities observed in recurrence time, time to metastasis and overall survival [5-7].

The resistance to a specific cytotoxic drug is ensured by the activity of efflux pumps that belong to the ABC-transporters super family. The most important in breast cancer are: PGP (P-glycoprotein), BCRP (Brest Cancer Resistance Protein), MRP1 (Multridrug resistance Protein 1) and MRP3 (Multidrug Resistance Protein 3). For example, in human cancer over-expression of PGP is linked to resistance to vinca alkaloids, anthracyclines, epipodophyllotoxins and also tubulin polymerizing drugs whereas MRP1 is responsible for resistance to vincristine and vinblastine, methothrexate, anthracyclines, etoposide, paclitaxel and irinotecan [8,9]. Unfortunately, expression of efflux pumps and their specific substrates have not been assessed in dogs yet. There is just one document published showing that BCRP can fully protect canine mammary cancer cells against doxorubicin [10].

That is why the aim of our study was to assess expression of PGP, BCRP, MRP1 and MRP3 in five canine mammary cancer cell lines and to investigate their role in cancer resistance to vinblastine, cisplatin and cyclophosphamide which are used in breast cancer treatment [11,12]. The examined drugs were selected based on the available data in the field of veterinary oncology. They presented positive results of cisplatin and cyclophosphamide treatment in canine mammary cancer [5,7] whereas vinblastine was selected due to its general tolerance [13]. Moreover, these anticancer agents belong to various groups, thus we used them to observe efflux pumps activity during treatment with drugs from different groups.

We also treated canine mammary cancer cells with siRNAs specific for these efflux pumps to knock-down their expression. Treatment of cancer cells by using classical inhibitors of efflux pumps often fails [8,9], thus we used a novel approach to reduce their activity.

Our results have pointed out that treatment of patients with the supportive use of the siRNAs specific for efflux pumps may help with improving results of chemotherapy. Despite the useful function of RNAi therapeutics for disease treatment, it, yet - still requires the development of clinically suitable, safe and effective drug delivery vehicles, there are some promising data from ongoing clinical trials that give hope for their practical application in the future [14].

## Methods

### Cell lines

The cell lines used for the study have been previously used in other published research [14-18]. Two canine mammary adenocarcinoma cell lines (CMT-W1, CMT-W2), anaplastic cancer cell line (P114), simple carcinoma cell line (CMT-U27) and spindle-cell mammary tumor cell line (CMT-U309) had been examined. Cells were cultured under optimal conditions: in RPMI-1640 medium enriched with 10% (v/v) heat-inactivated fetal bovine serum (FBS), penicillin-streptomycin (50 iU mL–1), and fungizone (2.5 mg mL–1) (reagents obtained from Sigma Aldrich, USA), in an atmosphere of 5% CO2 and 95% humidified air at 37°C.

### siRNA transfection

The siRNA transfection procedure used in canine mammary cancer cells was described in details in our previously published studies [19-21]. The cell density, transfection reagent toxicity and transfection efficacy were optimized according to the procedure described in our previous manuscript: [19]. The canine (*Canis lupus familiaris*) *pgp*, *bcrp*, *mrp1* and *mrp3* sequences were obtained from Gene Bank with accession numbers: NM_001003215, DQ222459.1, NM_001002971, XM_548204.2, respectively. The siRNA duplexes were designed by http://www.sigmaaldrich.com/life-science/custom-oligos/sirna-oligos/sirna-design-service.html. The results were confirmed using two independent algorithms: Dharmacon (OligoWalk) and Ambion and two duplexes were used for further experiments (obtained from Sigma Aldrich) (Table 1). For each gene silencing the mixture of both duplexes was used (30 pmol + 30 pmol). All the experiments with transfected cells were conducted 24–48 hrs after the transfection.

**Table 1:** Used siRNA sequences

**Gene**	**GenBank ID**	**First strand**	**Second strand**
*pgp*	NM_001003215	CGAACUGUUGUUUCUUUGA[dT][dT]	UCAAAGAAACAACAGUUCG[dT][dT]
		CUUCCGAACUGUUGUUUCU[dT][dT]	AGAAACAACAGUUCGGAAG[dT][dT]
*bcrp*	DQ222459.1	GCCCAGGAGUCAAUGUAAC[dT][dT]	GUUACAUUGACUCCUGGGC[dT][dT]
		CGAAUAAUACCUGUAGCUA[dT][dT]	UAGCUACAGGUAUUAUUCG[dT][dT]
*mrp1*	NM_001002971	GAAAGAGGCUCCCUGGCAA[dT][dT]	UUGCCAGGGAGCCUCUUUC[dT][dT]
		GGAGUAUUCAGAAACGGAG[dT][dT]	CUCCGUUUCUGAAUACUCC[dT][dT]
*mrp3*	XM_548204.2	GGCUAUGACGGAGAGCCAA[dT][dT]	UUGGCUCUCCGUCAUAGCC[dT][dT]
		UGUCUACGCUGCCUUGGGA[dT][dT]	UCCCAAGGCAGCGUAGACA[dT][dT]

*siRNA sequences used to* pgp, bcrp, mrp1 *and* mrp3 *genes silencing in canine mammary cancer cell lines*. *The canine (*Canis lupus familiaris*)* pgp, bcrp, mrp1 *and* mrp3 *sequences were obtained from Gene Bank with accession numbers: NM_001003215, DQ222459.1, NM_001002971, XM_548204.2, respectively. The siRNA duplexes were designed by*http://www.sigmaaldrich.com/life-science/custom-oligos/sirna-oligos/sirna-design-service.html*. The results were confirmed using two independent algorithms: Dharmacon (OligoWalk) and Ambion.*

### Reverse-transcriptase qPCR

Total RNA was isolated using a Total RNA kit (A&A Biotechnology, Poland) according to the manufacturer‘s protocol. Isolated RNA samples were dissolved in RNase-free water. The quantity of isolated RNA was measured using NanoDrop (NanoDrop Technologies, USA). The mean concentration of RNA was 173 ng/μl, and A260/280 ratio was between 1.8 and 2.0. The samples with adequate amounts of RNA were treated with DNaseI to eliminate DNA contamination. The samples were subsequently purified using RNeasy MiniElute Cleanup Kit (Qiagen). Finally RNA samples were analyzed on a BioAnalyzer (Agilent, USA) to measure final RNA quality and integrity. Only RNA with RIN (RNA Integrity Number) > 9 was used for the further analyses. Primers used to detect the expression of *pgp*, *bcrp*, *mrp1* and *mrp3* genes were designed using PRIMER3 software (free on-line access) and checked using Oligo Calculator (free on-line access) and Primer-Blast (NCBI database). The used sequences are listed in Table 2. *rps19* and *hprt* genes were used as non-regulated references for the normalization of target gene expression [22,23]. Quantitative RT-PCR was performed using fluorogenic SYBR Green and the Sequence Detection System, Fast 7500 (Applied Biosystems). Data analysis was carried out using the 7500 Fast System SDS Software Version 1.4.0.25 (Applied Biosystems, USA). The results were analyzed using comparative Ct method [24]. Relative transcript abundance of the gene equals ΔCt values (ΔCt = Ct^reference^ – Ct^target^). Relative changes in transcript were calculated as ΔΔCt values (ΔΔCt = 2^-ΔCt^). The experiment was conducted three times.

**Table 2:** Primers used for RT-qPCR

**Gene symbol**	**Forward primer**	**Reverse primer**	**Optimum annealing temp. (°C)**	**Optimum annealing time (sec)**
*pgp*	GCTTAACACCCGGCTCACAGAC	TAAGAAAGCGGCACCAATAGAAAT	72	10
*bcrp*	GAGCTCCTTGTGGTTGAGAA	AGGTGATGGTCATGAGGAGA	72	10
*mrp1*	TTTACTTTTCCCTCGTGCTG	TATTCAGGGACCAAAGGTCA	72	45
*mrp3*	AGGATGGACCTGATGACAGA	AACTTGGGAATCAGGAGACC	72	30
*hprt*	AGCTTGCTGGTGAAAAGGAC	TTATAGTCAAGGGCATATCC	59	6
*rps19*	CCTTCCTCAAAAAGTCTGGG	GTTCTCATCGTAGGGAGCAAG	61	10

Primer sequences used in this study and their annealing optimal temperature and time. The mRNA sequences of key genes were obtained from NCBI database. Primers were designed using PRIMER3 software (free on-line access) and checked using Oligo Calculator (free on-line access) and Primer-Blast (NCBI database). Primers sequences are listed in Table 1. *hprt* and *rps19* genes were used as non-regulated reference genes for normalization of target gene expression [22,23].

### Flow cytometry determination of rhodamine-123 accumulation

Examined cancer cells (5 × 10^6^ cells per ml) (control cells and cells treated with *pgp, bcrp, mrp1* and *mrp3* –specific siRNAs) were incubated for 1 h at 37°C in the 1 mmol of rhodamine-123 (obtained from Sigma Aldrich, Germany). After the incubation time, cells were washed twice, re-suspended in ice cold PBS and kept at 4°C in the dark until analysis in the flow cytometer. At least 50 000 cells per sample were counted and analyzed by flow cytometry (BD FACS Aria II, Becton Dickinson, USA). Cells shown in forward scatter and side scatter were gated and acquired through the fluorescence channel. The amount of fluorescence was plotted as a histogram within the gate. Data acquisition was performed using BD FACS Diva software (Becton Dickinson, USA) to determine mean fluorescence intensity values. This procedure has been published before [25]. Results represent the average of at least three independent experiments.

The overlay histogram was created using Flowing Software (Turku University, Finland), available at http://www.flowingsoftware.com.

### Cell viability assay (MTT-assay) and IC_50_ determination

Cell viability (metabolic activity of viable cells) was quantified by MTT assay. Cells were seeded into 96-well plate (Nunc Inc., Denmark) at the density which ensured their 50-70% confluence at the day of experiment and: 1) treated with vinblastine (at the following concentrations: 12.3, 36.9, 61.5, 123, 369, 615, 2 460, 4 920, 7 380 nmol), cisplatin (at the following concentrations: 46.5, 83, 465, 830, 4 650, 8 300, 46 500, 83 000 166 000 nmol) or cyclophosphamide (at the following concentrations: 425, 950, 1 900, 3 800, 19 000, 38 000 76 000 nmol) (all drugs obtained from Sigma Aldrich, Germany), 2) transfected with *pgp, bcrp, mrp1* and *mrp3* -specific siRNA and then treated with the anticancer drugs as given above, 3) treated with non-coding siRNA (scrambled control) and cyctotoxic drugs as given above. These concentrations of anticancer drugs were determined in course of our preliminary experiments (data not shown) and chosen as appropriate to determine IC50 doses in all of the examined cell lines.

Then, cells were incubated in 0.5 mg/ml tetrazolium salt MTT diluted in phenol red-free RPMI 1640 medium (Sigma Aldrich) for 4 hrs at 37°C. To complete solubilization of the formazan crystals, 100 μl of DMSO (Dimethyl sulfoxide, Sigma Aldrich) was added to each well. Cells viability was quantified by measuring photometric absorbance at 570 nm in multi well plate reader Infinite 200 PRO Tecan™ (TECAN, Mannedorf, Switzerland). All the samples were examined in triplicate, each experiment was conducted seven times (n = 21). Based on these results cytotoxicity was determined. It was expressed as a mean percentage decrease relative to unexposed control ± S.D. Control values were set at 100% viability. Cytotoxicity data were fitted to a sigmoidal curve and a four parameter logistic model was used to calculate IC50, which is the concentration of agent which reduces cell growth by 50% under the experimental conditions (increasing apoptosis, necrosis or causing block in cell cycle). This analysis was performed using GraphPad Prism 5.0 (San Diego, USA). This method of IC50 analysis has been previously published [26].

### Apoptosis assay

The Annexin V-FITC and propidium iodide (PI) dual staining was applied for apoptosis analysis. Control cells (1) and cells treated with (2) anticancer drug at IC50 dose and (3) transfected with *pgp*, *bcrp*, *mrp1* and *mrp3*-specific siRNA moreover treated with anticancer drug at IC50 dose were harvested by trypsinization. These cells, as well as the cells floating in medium (RPMI 1640 containing 10% FBS) were stained with an Annexin V Kit (Becton Dickinson, USA) according to the manufacturer’s protocol. The cells then were analyzed by flow cytometry (BD FACS Aria II, Becton Dickinson, USA) within 1 hr after staining. Early apoptotic cells with exposed phosphatidylserine but intact cell membranes bound to Annexin V-FITC however excluded PI. Cells in late apoptotic stages were labeled with both Annexin V-FITC and PI, whereas necrotic cells were labeled with PI only. All samples were assayed in triplicate. The experiment was conducted at least twice.

### Statistical analysis

The analysis for statistical purposes was conducted using Prism version 5.00 software (GraphPad Software, USA). The one-way ANOVA and Tukey HSD (Honestly Significant Difference) post-hoc test were applied as well as regression analysis. Determination of IC50 test has been used. The p-value <0.05 was recognized as significant, whereas, p-value <0.01 and p-value <0.001 as highly significant. The data was expressed as means +/− S.D. For molarity calculations, the Molarity Calculator (GraphPad, USA) on-line platform was used.

## Results

### PGP, BCRP, MRP1 and MRP3 are expressed in canine mammary cancer cells and their expression level changes due to anticancer drug *in vitro* treatment

RT-qPCR analysis revealed that all of the examined cell lines express *pgp*, *bcrp*, *mrp1* and *mrp3* (Table 3). The *pgp* expression ranged between 3.50 in CMT-U27 cell line and 17.85 in CMT-W1 cell line. The *bcrp* expression was the lowest in CMT-U309 cell lines (11.86) whereas the highest in CMT-W2 cell line (36.00). The highest expression of *mrp1* was observed in CMT-W2 cell line (24.02) whereas the lowest expression was noticed in CMT-U27 cell line (7.50). CMT-W2 cell line exposed the lowest expression of *mrp3* (11.35). The highest expression of *mrp3* was detected in CMT-U309 cell line (23.28).

**Table 3:** **Expression of *pgp, bcrp, mrp1 *and *mrp3 *in various experimental conditions**

**Efflux pump**	**Sample**	**Relative expression in cell lines**				
		**CMT-U27**	**CMT-U309**	**P114**	**CMT-W1**	**CMT-W2**
**PGP**	**ctrl**	3.50	9.20	5.00	17.85	16.42
	***pgp *siRNA**	0.45***	0.10***	0.50***	0.80***	0.16***
	**non-coding siRNA**	3.44	9.10	5.10	7.750	16.31
	**vinblastine**	9.50***	16.20***	14.70***	19.56***	21.52***
	**cisplatin**	8.95***	35.20***	31.00***	36.08***	26.16***
	**cyclophosphamide**	0.09***	2.30**	0.22***	0.08***	2.87***
**BCRP**	**ctrl**	19.84	11.86	23.52	23.70	36.00
	***bcrp *siRNA**	17.15**	2.77***	2.77***	10.46***	0.00***
	**non-coding siRNA**	19.00	11.20	22.90	23.00	36.18
	**vinblastine**	14.84*	3.80***	11.80***	12.30***	13.24*
	**cisplatin**	30.50***	22.16***	40.34***	36.57***	37.23*
	**cyclophosphamide**	23.32***	33.53*	29.23***	24.50*	41.72**
**MRP1**	**ctrl**	7.50	9.40	16.50	20.10	24.02
	***mrp1 *siRNA**	0.10***	0.60***	0.23***	0.20***	0.61***
	**non-coding siRNA**	7.20	6.62	20.12	20.91	24.21
	**vinblastine**	9.70***	7.00***	9.76***	35.01*	30.20***
	**cisplatin**	15.70***	19.34**	21.84*	30.61***	26.20**
	**cyclophosphamide**	2.50**	0.09***	3.32***	↓0.40***	6.04**
**MRP3**	**ctrl**	14.40	23.28	13.84	17.70	11.35
	***mrp3 *siRNA**	1.00***	0.00***	4.84***	13.90**	7.15**
	**non-coding siRNA**	13.87	21.99	13.09	17.10	10.99
	**vinblastine**	14.20	21.97	13.12	17.50	9.78
	**cisplatin**	15.60*	32.20***	15.80*	21.30***	14.09*
	**cyclophosphamide**	14.99	24.09	14.25	18.01	11.72

Expression of efflux pumps in canine mammary cell lines in control conditions, after scrambled siRNA treatment and *pgp, bcrp, mrp1* and *mrp3* -specific siRNA treatment as well as after treatment with vinblastine, cisplatin and cyclophosphamide at IC50 doses. p < 0.05 was marked as*, p < 0.01 was marked as **, and p < 0.001 was marked as ***. One-way ANOVA followed by Tukey HSD post-hoc test were applied. Increase in expression was marked as red text, decreased in expression was marked as green text, whereas no change in transcript level was marked as black text.

Treatment of cancer cells using scrambled siRNA did not cause any significant effect on examined gene expression (Table 3), whereas treatment of these cells with *pgp*, *bcrp*, *mrp1* or *mrp3* -specific siRNAs caused significant decrease in transcript level of targeted genes (the mostly significant effect was noticed in case of *pgp* and *mrp1*) (Table 3). The efficacy of silencing reaction was very high, as there was observed 88-99% decrease in *pgp* transcript level, 14-100% in *bcrp*, 94-99% in *mrp1* and 22-100% in *mrp3* expression (Table 3).

Interestingly, we observed that *pgp* expression increased significantly in all the examined cell lines after viblastine and cisplatin treatment at IC50 doses, whereas decreased after cyclophosphamide treatment at IC50 dose (Table 3). Vinblastine treatment increased *mrp1* expression in all of the examined cell lines with exception of CMT-W2 cell line where its expression increased also after treatment with cyclophosphamide at IC50 dose (Table 3). In case of *bcrp*, we observed increased tendency in its expression after cyclophosphamide treatment and decreased in its expression after vinblastine treatment at IC50 dose (Table 3). We are as well able to point out the increase in *mrp3* expression in all the examined cell lines after treatment with cisplatin at IC50 dose (Table 3).

### Cancer cell treatment with *pgp, bcrp, mrp1* and *mrp3* –specific siRNA decreases efflux pumps activity enhancing rhodamine-123 accumulation

**Figure 1:**
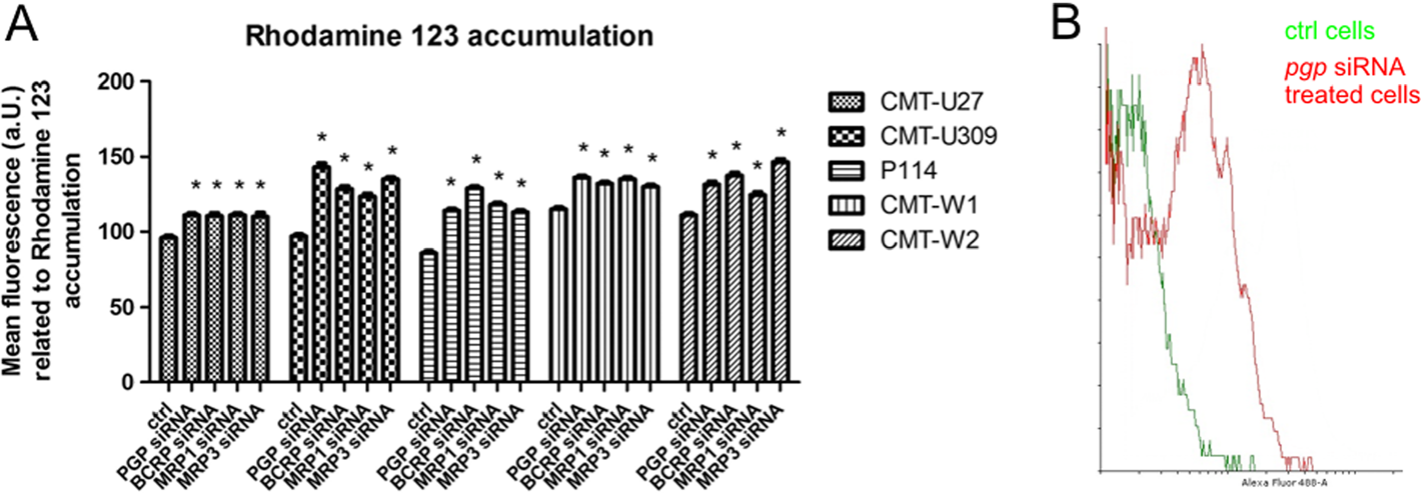
**Activity of PGP, BCRP, MRP1 and MRP3. A**.) Graph of mean fluorescence related to rhodamine-123 accumulation inside cancer cells obtained using FACS Aria II (Becton Dickinson, USA). Error bars refer to S.D. p < 0.05 was marked as *. One-way ANOVA followed by Tukey HSD post-hoc test was applied. **B**.) Representative overlay histograms of rhodamine-123 accumulation in CMT-U309 canine mammary cancer cells (control, markered as green), and treated with *pgp* -specific siRNA (markerd as red). The overlay histograms were created using Flowing Software (Turku University, Finland), http://www.flowingsoftware.com.

The rhodamine-123 efflux assay was conducted to confirm our results of gene expression silencing and their functional inhibition. It showed that after knock-down of *pgp*, *bcrp*, *mrp1* and *mrp3* expression level, the rhodamine-;123 accumulation significantly increased and that shows decreased activity of the efflux pumps (Figure 1A, B). Treatment of examined cells with scrambled siRNA did not cause any significant effect (data not shown). We observed that siRNA treatment of CMT-U27 caused significant but slight effect on rhodamine-123 accumulation (mean fluorescence related to rhodamine-123 accumulation in control cells was 97, whereas in siRNA treated cells it was between 108 and 110). In other cell lines the effect was higher (Figure 1), for example in P114 cell line the mean fluorescence related to rhodamine-123 accumulation in control cells was only 87 (the lowest, comparing to other cell lines) however, after the treatment with *bcrp*-specific siRNA is was 128 (Figure 1). The highest effect of rhodamine-123 accumulation after siRNA treatment was observed in CMT-W2 cell line (148 after *mrp3*-specific siRNA treatment, 111 in control conditions).

### Cancer cell treatment with siRNA specific to efflux pumps decreases drug-resistance

**Figure 2:**
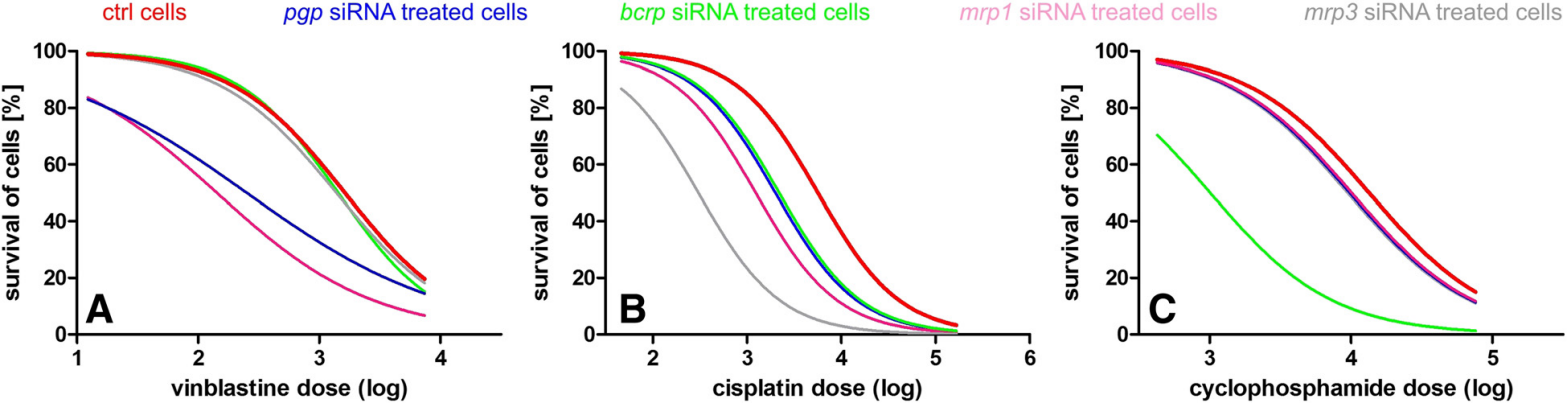
**Survival curves of CMT-U27 cells.** Survival curves of CMT-U27 cells treated with increasing doses of vinblastine (**A**), cisplatin (**B**) and cyclophosphamide (**C**). IC50 doses were determined in control conditions and after *pgp*, *bcrp*, *mrp1* and *mrp3* silencing. IC50 was calculated using GraphPad Prism 5.0 (San Diego, USA).

The MTT assay of cells viability after treatment with increasing doses of anticancer drugs showed significant variations in IC50 doses between the examined cell lines. All IC50 doses are listed in Table 4. Vinblastine IC50 dose in CMT-U27 cell line was 1 588 nmol, whereas after PGP and MRP1 knock-down it was only 203 nmol and 144 nmol, respectively (Table 4, Figure 2A). In CMT-W2 cell line vinblastine IC50 was 9 434 nmol, whereas after *pgp* and *mrp1* silencing it was 1264 nmol and 1248 nmol, respectively (Table 4). We showed that after *pgp* and *mrp1* silencing IC50 was significantly lower in all the examined cell lines ranging 203–1391 nmol and 144–1248 nmol, respectively (Table 4). Significant variations were also observed in case of IC50 doses of cisplatin – the lowest IC50 dose was in CMT-U27 cell line (5 669 nmol) (Figure 2B), whereas the highest was in P114 cell line (42 944 nmol) (Table 4). Cisplatin IC50 was significantly decreased in all the examined cell lines after silencing of *pgp*, *bcrp*, *mrp1* and *mrp3* (Table 4). Regarding cyclophosphamide, we also noticed significant variations in IC50 values. It was the lowest in CMT-W1 cell line (8 679 nmol), when on the other hand the highest in CMT-W2 cell line (25 698 nmol) (Table 4). Treatment of cells with *bcrp*-specific siRNA decreased cyclophosphamide IC50 in all of the examined cell lines (ranging between 765 and 5 705 nmol) (Table 4, Figure 2C).

**Table 4:** IC50 doses of anticancer drugs in control conditions and after efflux pumps silencing

**Anticancer drug**	**Condition**	**IC50 in examined cell lines [nmol]**				
		**CMT-U27**	**CMT-U309**	**P114**	**CMT-W1**	**CMT-W2**
**vinblastine**	**ctrl**	1 588	2 472	2 744	8 072	9 434
	**pgp siRNA**	203***	464***	208***	1 391***	1 264***
	**bcrp siRNA**	1 497	2 417	2 572	8 002	7 346
	**mrp1 siRNA**	144***	772**	756**	1 747***	1 248***
	**mrp3 siRNA**	1 330	2 406	2 561	7 433	8 482
**cisplatin**	**ctrl**	5 669	12 034	42 944	41 288	9 536
	**pgp siRNA**	2 203**	8 613**	5 000***	32 663***	6 707**
	**bcrp siRNA**	3 300**	8 540**	17 699***	11 228***	8 177*
	**mrp1 siRNA**	1 339**	10 090*	20 348***	29 132***	5 695**
	**mrp3 siRNA**	303***	7 301**	13 565***	11 163***	7 177*
**cyclophosphamide**	**ctrl**	9 599	12 542	10 340	8 679	25 698
	**pgp siRNA**	8 432	11 755	10 242	7 912	15 443
	**bcrp siRNA**	799***	2 086***	5705**	2 463**	765***
	**mrp1 siRNA**	8 762	11 307	9 955	7 247	7 000
	**mrp3 siRNA**	8 802	12 278	10 167	6 932	1 130

IC50 doses [nmol] of anticancer drugs: vinblastine, cisplatin and cyclophosphamide in canine mammary cancer cell lines in control conditions and after silencing of efflux pumps expression. p < 0.05 was marked as *, p < 0.01 was marked as **, and p < 0.001 was marked as ***. One-way ANOVA followed by Tukey HSD post-hoc test was applied.

### Cancer cell treatment with anticancer drug and siRNA specific to efflux pumps increases apoptosis

The Annexin V assay was used to examine the influence of *pgp*, *bcrp*, *mrp1* and *mrp3* expression knock-down on apoptosis induced by vinblastine, cisplatin and cyclophosphamide (comparing to anticancer drug given as a single agent at IC50 dose). Due to any changes in cells viability based on transfection procedure were observed in MTT assay, this kind of control has been omitted in apoptosis assay. We used similar approach in our previous study [19].

**Figure 3:**
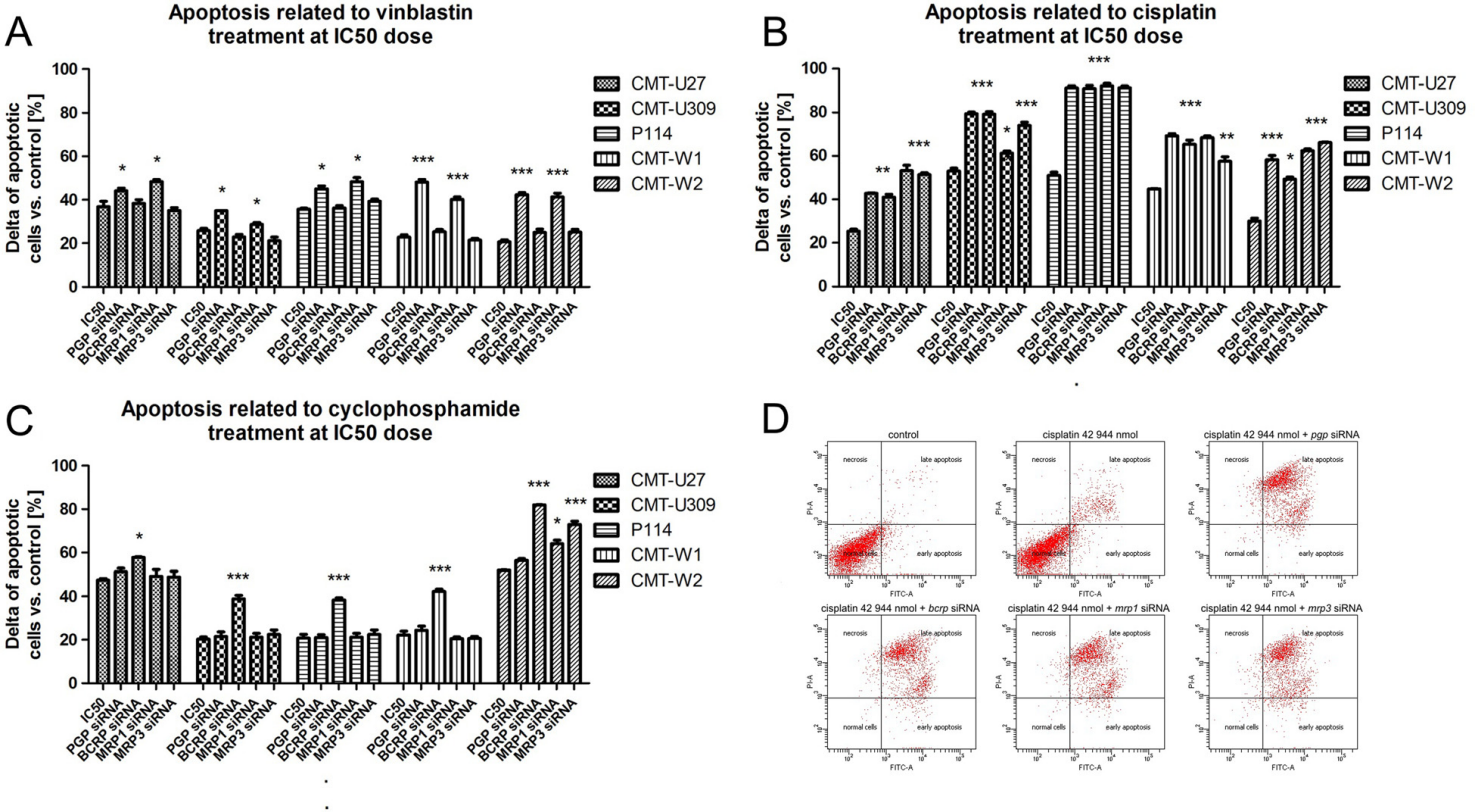
**Apoptosis related to treatment with anticancer drugs.** The number of apoptotic cells due to treatment with vinblastine (**A**), cisplatin (**B**) and cyclophosphamide (**C**) at IC50 doses in control conditions and after efflux pumps silencing in canine mammary cancer cell lines (CMT-U27, CMT-U309, P114, CMT-W1 and CMT-W2) assessed by the Annexin V/PI test (BD Bioscience, USA). All values were calculated versus control conditions (spontaneous apoptosis in these cell lines). The numbers of apoptotic cells are represented as a percentages of Annexin V-positive cells (obtained with FACS Aria II Becton Dickinson). The experiment has been conducted in three replicates. Error bars refer to S.D. p < 0.05 was marked as *, p < 0.01 was marked as ** and p < 0.001 was marked as ***. One-way ANOVA followed by Tukey HSD post-hoc test was applied. (**D**) The representative cytograms of P114 cell line double stained with Annexin V-FITC and propidium iodide (PI). The cytograms show P114 cells in control conditions, after cisplatin treatment at IC50 dose and after *pgp, bcrp, mrp1* and *mrp3* -specific siRNA treatment followed by cisplatin treatment at IC50 dose. On the cytograms are showed normal, early apoptotic, late apoptotic and necrotic cells. Left bottom quadrant shows normal cells, top left quadrant shows necrotic cells (stained with PI only; damaged cell membrane but no phosphatydilserine exposure), right bottom quadrant shows the early apoptotic cells (stained with Annexin V only; intact cell membrane) and top right quadrant shows cells in late stage of apoptosis (stained with Annexin V and PI - phosphatydilserine exposure and damaged cell membrane).

Annexin V analysis revealed that treatment of cells using *pgp* and *mrp1* -specific siRNA together with vinblastine (at IC50 dose) significantly increased number of apoptotic cells, compared to apoptosis caused by anticancer drug which was given as a single agent (at the IC50 dose as well) (Figure 3A). The most significant effect was pointed out in CMT-W1 cell line: 25.35% and 17.45% increase in number of apoptotic cells after *pgp* and *mrp1* silencing and vinblastine treatment, respectively (Figure 3). Similarly, in CMT-W2 cell line 21.78% and 20.88% increase in number of apoptotic cells was shown after *pgp* and *mrp1* silencing and vinblastine treatment, respectively (Figure 3).

In case of cisplatin treatment at IC50 dose, an increase in number of apoptotic cells was noticed in each cell line after silencing of all the efflux pumps: *pgp*, *bcrp*, *mrp1* and *mrp3*. The mostly significant result was observed in P114 cell line, where 40%, 41%, 42% and 40% increase in number of apoptotic cells (p < 0.001) was visible*,* respectively (Figure 3B, D). In CMT-U309 cell line the highly significant (p < 0.001) effect on apoptosis was as well as in case of cisplatin treatment in cells transfected with siRNA specific to: *pgp*, *bcrp* and *mrp3* (26.45%, 26.18% and 21.05% increase in number of apoptotic cells, respectively), whereas significant effect (p < 0.05) was observed in cells transfected with *mrp1* -specific siRNA (8.25% increase in number of apoptotic cells) (Figure 3B).

In all the examined cell lines increase in number of apoptotic cells related to cyclophosphamide treatment at IC50 dose was undoubtedly visually recognized after *bcrp* knock-down (Figure 3C). Additionally, in CMT-W2 cell line the significant increase in number of apoptotic cells was observed due to cyclophosphamide treatment following transfection with *bcrp*, *mrp1* and *mrp3* -specific siRNA (30%, 12.1% and 21%, respectively).

## Discussion

Cancer cells retain the important mechanism of self-protection through the activity of multiple drug resistance transporters. The multidrug resistance is frequently associated with over-expression of two or more membrane pumps that efflux anticancer drug from the cytoplasm. This protects tumor cells against the drug effects and its correlated molecular processes [8]. Expression of efflux pumps is usually higher in tumors that originate from tissues that normally show their expression and it is always higher in the tumors than in normal cells [8]. Unfortunately, the expression and role of proteins that mediate drug resistance in canine cancer cells has not yet been recognized. As increased efflux is such a significant contributor to a multidrug resistance in cancer cells, current research is aimed at blocking or inhibiting this specific mechanism. That is why the aim of our study was to assess expression of four the most important efflux pumps: PGP, BCRP, MRP1 and MRP3 in canine mammary cancer cells as well as to investigate their role in resistance to: cisplatin, cyclophosphamide and vinblastine. Because treatment of cancer cells with the use of classical inhibitors of efflux pumps often fails [8,9], we used a novel approach, that is: specific RNAi to knock-down their expression.

Examination of each efflux pump expression in control conditions and after treatment with anticancer drug was also quite a novel approach. We observed significant differences in *pgp* transcript level in control conditions and after treatment with anticancer drug. In all of the examined cell lines the *pgp* expression increased due to vinblastine and cisplatin treatment whereas decreased due to cyclophosphamide treatment (Table 3). It ought to be outlined that these results are in accordance with IC50 doses (Table 4). In case of cisplatin treatment, expression of all four efflux pumps increased, and thus, any relationship between their transcript level and IC50 doses was very complicated to calculate (Tables 3 and 4). We also observed that cyclophosphamide treatment caused increase in *bcrp* expression in all of the examined cell lines. These results are in accordance with previous findings that cytotoxic drugs can initiate or increase expression of efflux pumps in cancer cells [27]. Moreover, the expression level of these efflux pumps correlates with response to anticancer therapy and patient follow up [28]. Based on our results, we have come up with the conclusion that examination of efflux pumps expression before initiation of chemotherapy could help to predict response to anticancer drugs and could be helpful in assessment of their proper doses. However, more studies in this field are required.

The RNAi gene silencing was highly successful (comparing to our previously published studies [19,21]), reaching even 100% of gene knock-down in case of *bcrp* in CMT-W2 cell line and *mrp3* in case of CMT-U309 cell line. The results of gene knock-down were confirmed by the assay with rhodamine-123 efflux. To assess whether efflux pumps silencing has an influence on cancer cell susceptibility to cytostatic drugs, the IC50 doses were determined in transfected cells (Table 4, Figure 2). Our results showed that vinblastine IC50 doses significantly decreased in all the examined cell lines after *pgp* and *mrp1* silencing (Table 4). Examination of cisplatin IC50 in transfected cells pointed that it was significantly lower in all of them when compared to control cells (Table 4). In case of cyclophosphamide, *bcrp* siRNA treatment significantly decreased IC50 dose in all of the examined cell lines (Table 4).

Comparison of IC50 doses of anticancer drugs given *in vitro* and their maximum possible plasma concentrations showed that in case of vinblastine the plasma concentration was much lower than the IC50 doses [29]. It means that the examined cancer cell lines are vinblastine-resistant and therefore this anticancer drug may be ineffective in canine mammary cancer treatment. However, more studies in this field should be conducted. In case of cisplatin, the IC50 doses in the three examined cell lines (CMT-U27, CMT-U309 and CMT-W2) were lower than the reachable plasma concentrations [30]. However, after *bcrp, mrp1* or *mrp3* knock-down, the cisplatin IC50 doses were similar or lower than the maximum possible plasma drug concentration in all of the examined cell lines. It means that knock-down of efflux pumps expression during chemotherapy can reverse cancer drug-resistance. The IC50 doses of cyclophosphamide in all of the examined cell lines were significantly lower than its maximum possible plasma concentration [31]. However, in all the cases given above, targeting of ABC efflux pumps using siRNA significantly decreased IC50 doses of anticancer drugs. These results were also confirmed by apoptosis assay (Figure 3).

In summary: knock-down of efflux pumps expression during chemotherapy could allow decreased doses of anticancer drugs thereby reducing the risk of their side effects. However, more studies in this field are required as well as large clinical trials to prove this hypothesis.

Our research is a completely new approach in the field of veterinary oncology. The results show that targeting of ABC-efflux pumps using RNAi can be promising strategy in cancer treatment. However, these *in vitro* studies should be confirmed using *in vivo* animal models.

## Conclusions

The most important findings of our study are: (1) the expression level of efflux pump reflects the resistance to specific anticancer drug thus it should be assessed before the initiation of chemotherapy; (2) in canine mammary cancer PGP and MRP1 are responsible for vinblastine resistance, PGP, BCRP, MRP1 and MRP3 are cisplatin transporters whereas BCRP is a transporter of cyclophosphamide; (3) treatment of cells using siRNA targeting efflux pumps significantly increases cancer susceptibility to anticancer drug and allows to decrease the effective dose of anticancer drug.

## Competing interests

The authors declare that they have no competing interests.

## Authors’ contributions

KP research design, experimental design, IC50 determination, FACS analyses, rhodamine-123 accumulation assay, siRNA transfections, JM apoptosis analysis, KM apoptosis analysis, TM manuscript preparation, MK research design, experimental design, FACS analyses, siRNA transfection, RT-QPCR, manuscript preparation. All authors read and approved the final manuscript.
